# Rethinking microplastic cleanup: sustainable bioremediation compared to conventional physical-chemical methods

**DOI:** 10.1039/d6ra02881k

**Published:** 2026-05-27

**Authors:** Sudeshna Chell, Uttiya Dey, Kousik Das, Pankaj Kumar

**Affiliations:** a Department of Environmental Science and Engineering, SRM University-AP Amaravati-522240 Andhra Pradesh India kousik.d@srmap.edu.in; b Department of Environmental Science, Kunwar Singh College Laheriasarai Darbhanga-846001 Bihar India uttiya.1988@gmail.com; c Institute for Global Environmental Strategies Hayama 240-0115 Kanagawa Japan

## Abstract

Microplastics (MPs) (<5 mm) are hydrophobic and can't be easily degraded. These particles accumulate due to the excessive discharge of plastic waste from domestic and industrial sources, along with several toxic compounds attached to their surface, are also being deposited in environmental matrices, hampering the sustainable environment and human well-being. In the present scenario, to maintain sustainability, there is an urgent need to develop sustainable removal technologies. Physical and chemical treatment of MP removal, such as membrane filtration, density separation, adsorption, coagulation, and photo-catalysis, works effectively, but it is limited by sustainable energy demand, high cost, and secondary pollutants. Ultrafiltration membranes composed of polyether sulphone (PESP) are able to 91–96% removal efficiency for PE, PVC, while Zn–Al layered double hydroxide granules removed up to 96% of nano plastic debris (NPDs). In biological remediation, *Aspergillus tubingensis* degraded approximately 90% of polyurethane (PUR) through esterase and lipase activities at 37 °C under aerobic conditions within 60 days, whereas *Phanerochaete chrysosporium* removed up to 31% of PVC through peroxidase under acidic aerobic conditions. The primary objective of this study is to critically evaluate physical-chemical MP removal technologies, in comparison with bioremediation, which is more sustainable, cost-effective, eco-friendly process. Microorganisms and their enzymes degrade MPs by breaking them through fragmentation, de-polymerization and mineralization. MP degradation by mixed-culture, including bacteria, fungi, and microalgae, is more sustainable method to mitigate the emerging MP contamination. This review highlights the advantages of bioremediation over conventional processes, emphasizing its potential for large-scale application in MP management.

## Abstract 2

Integrated sustainable microplastic remediation techniques and circular economy framework.

## Graphical abstract

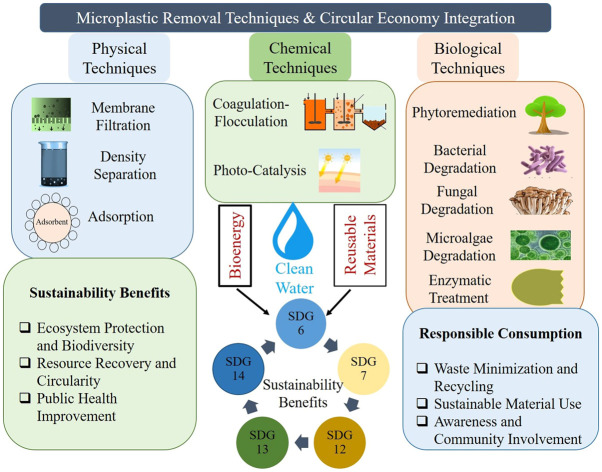

## 1. Introduction

The usefulness of plastic products has increased massively from the time of their invention due to some unique properties it possesses, like high strength, low weight, durability, thermal and water resistance, inexpensive production process *etc.*^1,2^ On the other hand, it is also creating alarming concern as this persistent pollutant poses great threat to the natural environment because plastics are recalcitrant to the natural degradation process and centuries may be required for the plastic degradation process.^3,4^ Generally high molecular weight polymers are known as plastics which are derived from natural gas and petroleum. Several different compounds are added up to comprise plastic molecules which include basic compounds like monomers, oligomers and polymers along with additives like antioxidants, heat stabilizers, pigments and plasticizers.^5^ MPs vary widely according to their shape, size and chemical composition. Beads, fiber, filaments, foam and fragments are different types of MPs according to the shape and size. Accordingly, polyethylene (PE), polypropylene (PP), polystyrene (PS), polyethylene terephthalate (PET), polyvinyl chloride (PVC), low density polyethylene (LDPE) and high-density polyethylene (HDPE) are the most used synthetic plastics which constitute 90% of the total plastic production in the entire world.^6,7^ Among all these plastic types, polyethylene and polypropylene contribute for almost half of the total plastic products throughout the world.^8^

Globally plastic production has increased to 1.3 million tons to 359 million tons up to 2018.^9^ Among all the plastic products manufactured and comes to the market to be used in different sectors like domestic, commercial *etc.* 70% ends up in the natural environment as waste products,^10^ only 10% being recycled and around 14% incinerated.^11^ In addition, due to recent COVID-19 outbreak, the production and disposal of face masks, personal protection kits and other medical supplies have significantly increased which have added large volume of plastic waste to the environment.^2^ Recently there is another new addition to the existing plastic pollution, microplastics (MPs) which are tiny plastic particles, having the size range of about 5 mm to 100 µm in diameter.^12–14^ These particles are strong hydrophobic in nature, have larger surface area and the eco-friendly degradation process is even much lower in comparison with the macro plastics.^1^ Primary MPs come to the environment from the industries which use small plastic beads, particles or powder as raw materials, like plastic toy and utensils factories, packaging industries, some personal care products, cosmetics and pharmaceutical factories *etc.* Which directly throw their waste into the natural ecosystem without any treatment.^15–17^ Globally the production rate of MPs in these sectors has increased to 1.038 billion (2017–2019) which is expected to increase further to be double in the next decades.^18^ On the other hand, secondary microplastics are the degraded forms of large plastic products. Physical (physical abrasion by wind and ocean wave, atmospheric turbulence), chemical (action of UV radiation, oxidation, photo-degradation) and biological (degradation by tiny insects and microbes) forces help in breakdown and fragmentation of plastic products into smaller particles.^19,20^ Because of very low weight, MPs can be transported through water and air also, so they are found in a vast range of ecosystems from deep sea sediment to the polar region.^21^ Because of their tiny diameters which sometimes are indistinguishable in naked eyes, the MPs become more hazardous to humans as well as to the entire environment than that of larger non-degradable plastic wastes.^22^ These tiny particles can cause serious harm to the organisms when they ingest the MP particles by mistaking them as food which leads to false hunger satisfaction, digestive disorders and intestinal abrasions along with some indirect effects like induced oxidative stress, reduced growth, physiological and reproductive disorders, neurotoxic effects, DNA damage *etc.*^23,24^ It also gets into the human body through food chain resulting in harmful health effects.^25^ These particles also act as transporter of harmful chemicals into the tissues of different animals because apart from the core chemical compounds, plastic can also absorb toxic chemicals like persistent organic pollutants, heavy metals onto their surface which eventually makes them a toxic hub.^23,26^ The MPs having larger surface area tend to accumulate more toxic substances compared to large plastics.^2^ MPs can also act as carrier of various microorganisms including pathogenic organisms which eventually can cause major diseases outbreak.^27^ Plastic pollution and the life cycle of plastic products as well as MPs have negative impacts on biodiversity of an area, even one of the major causes for the loss of biodiversity.^26^ Eradication of this critical problem should be one of the major objectives of the scientific community. As MP is a recent emerging contaminant, most of the research are involved in the determination and chemical characterization of MP particles in different environmental media rather than remove it, because for the removal purpose, baseline data about the pollution load should be reported first.

Researchers started to work on the removal of MPs through various ways recently including physical chemical as well as biological processes. Common physical removal processes are thermo-degradation, photo-degradation which takes long time for the degradation of MPs,^28,29^ adsorptions which creates another by product as well as the removal efficiency is also very low like the process density separation also. Chemical approaches in which plastic particles can be melted down by using organic solvent like acetone,^30^ but in this case the organic solvent is a toxic substance itself. In other chemical approaches of MP removal like coagulation, oxidation-reduction and photocatalysis, the main issue is about high energy consumption and the production of secondary pollutants.^31^ On the other hand, depolymerization of waste plastics into monomers through microbial or enzymatic degradation is one of the most promising strategies for either recycling or mineralization of the polymers into carbon-di-oxide, water and other new byproducts of higher value.^32,33^ Plastic biodegradation by microorganisms is a very complex process in which first the particular microorganism secretes some extracellular enzymes which attaches to the plastic surface and hydrolyze them first into short intermediate polymer molecules and finally into monomers which are easily assimilated by the microorganisms that uses them as a carbon source and the microorganism ultimately release CO_2_ into the environment.^4^ More than 100 plastic degrading microorganisms, both bacteria and fungi have been reported in past few years which are effectively capable of metabolizing different types of polymers mostly in the laboratory.^34^ If these plastic degrading microbes can be modified through genetic engineering and their efficiency can be enhanced by means of microbial biotechnology it will provide enormous societal benefits by providing improved recycling procedure which will produce carbon source and valuable alkane products and on the other hand it will help to reduce the plastic waste burden from the environment.^4^ The review covers physical, chemical, and biological remediation strategies for microplastic and nanoplastic removal, while maintaining a primary focus on microbial-mediated biodegradation approaches. There are a very few studies which are focusing the bioremediation of MPs either by enzymes or microorganisms. So, from the above background study, we will be discussing the biodegradation strategies of microplastics by different types of microorganisms in this present study.

## 2. Bibliometric analysis

**Fig. 1:**
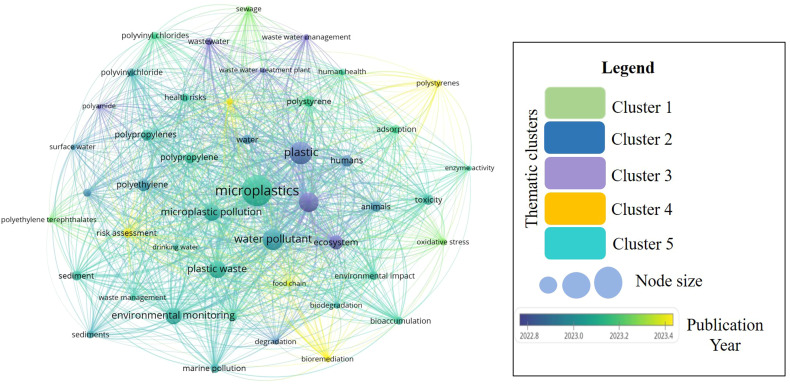
Integrated bibliometric keywords network highlighting major thematic clusters and research trends associated with remediation of microplastic studies.

The bibliometric analysis of 353 Scopus-indexed publications (2020–2025) on “Remediation of Microplastics” reveals a structured evolution of scientific inquiry in this domain generated by VOS-software. The keyword ‘microplastics’ as the central node, strongly co-occurring with ‘plastic pollution’, ‘ecotoxicology’, ‘water pollution’, and ‘biodegradation’, indicating the convergence of environmental assessment and remediation methodologies. The legends describe the node size as the frequency of keyword occurrence, the connecting lines as the co-occurrence relationships between keywords, and the different colors as thematic research clusters. Different colors in the map represent thematic clusters generated automatically by this software, indicating groups of closely related research topics such as different polymer types, risk assessment, health risk, environmental monitoring, biodegradation, and bioremediation. Cluster 1 mainly represents environmental contamination and wastewater-related studies, while Cluster 2 focuses on polymer composition, water pollution and distribution of microplastic, Cluster 3 refers to the core research domain linking microplastics with ecological and human health risk, whereas Clusters 4 and 5 denotes toxicological impacts, adsorption behaviour, degradation, and remediation strategies. In this network map, each circle node represents a keyword, and the size of the node indicates the frequency of occurrence of that keyword within the selected publications. Larger nodes represent highly studied and the connecting lines between nodes indicate a co-occurrence relationship, showing how frequently these keywords appeared together in the same publications. Network and cluster analyses (Fig. 1) identify four major research areas: (i) environmental monitoring, which includes detection methods. (ii) Toxicological impacts, focusing on bioaccumulation, effects on organisms and human health, (iii) bioremediation and degradation, examining microbial communities, enzyme-driven breakdown, and related pathways, and (iv) polymer characterization. It shows a clear shift from early research (2022–2023) that emphasized occurrence, fate, and risk assessment to more recent studies (2023–2025) that focus on sustainable, energy-efficient, and scalable remediation technologies. This trend indicates a gradually advancing field moving toward practical, innovative solutions for microplastics pollution.

## 3. Current technologies for the removal of microplastics and their demerits

**Fig. 2:**
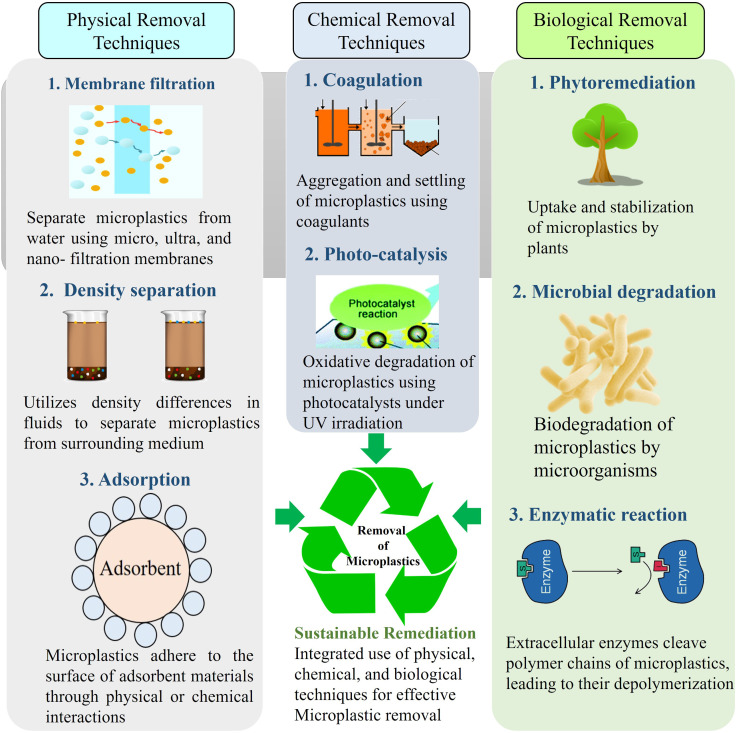
Innovative Physical, Chemical, and Biological treatment for the removal of microplastics from environmental media.^31,36,37^

Microplastics being among the most recent emerging contaminants experiments on the removal technologies are absolutely in preliminary stage. Most of the research are focusing on the determination of the contamination load in different media and the development of efficient methods for qualitative as well as quantitative analysis. Lack of effective technology for the determination of MP concentration is also a hurdle for the development of removal techniques. Also, identification of different types of MPs according to the shape, size, color as well as chemical characteristics are also one of the challenges faced for the elimination of MP pollution.^35^ Most of the removal experiments till date are done in the laboratory scale by mixing commercially available MP beads or particles with deionized water where no other contaminants present. But in the natural condition there are high possibilities of multiple contaminants to interfere in the entire procedure.^31^ The existing technologies for the MPs removal can be broadly categorized into physical, chemical and biological (Fig. 2). In physical methods the MPs are separated from the media and the concentration is reduced to an acceptable limit as used in the waste water treatment plants but the MP particles are retained in the sludge which most of the times is used in the agricultural field as manure, leading to the contamination of another media, the agricultural soil.^31,36^ In the chemical process also the use of different chemicals to degrade the plastic can in turn create problem to the natural environment. On the other hand, the bioremediation of MPs refers to the use of biological agents such as microbes and certain plants which can convert the polymers into simple monomers which are less harmful and easily degradable and sometimes the MPs get completely mineralized through enzymatic degradation.^37^

## 4. Physical process for the removal of microplastics

### 4.1. Membrane filtration

**Fig. 3:**
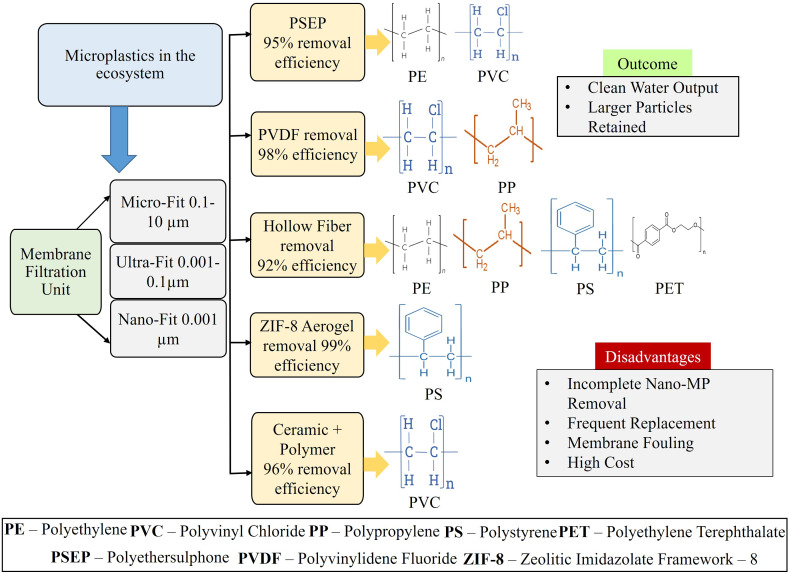
Schematic illustration of membrane filtration pathways for the removal of microplastics, showing filtration ranges, removal efficiencies, polymer types, treatment outcomes, and associated disadvantages.^39–41^

Filtration is the most widely used technology for the removal of MPs from water used in most of the wastewater treatment plants (WWTP) and drinking water treatment plants (DWTP), the retained concentrated fraction still contains microplastics and pollutants. The efficiency of the process mainly depends on the size of the MPs and the pore size of the filtration membrane, the larger particles are rejected by the membrane to pass through and enter the clean water system. The smaller the pore size, the more effective the filtration unit becomes. The effectiveness of the membrane filtration process depends on the characteristics of the membrane, *trans*-membrane pressure gradient, filtration time, rate and velocity of the solution flow and size of the MPs.^9^ The main working principle of this process is the pressure driven separation of two different phases on the either sides of the membrane.^38^ The process can be categorized depending on the size of the MPs like micro-filtration, nano-filtration, and ultra-filtration (Fig. 3).^39–41^ In addition, with the conventional granular filtration processes like sand filtration and activated charcoal filtration,^31^ and with the development of new technologies, different kind of advanced and high efficiency membrane have been evolved in recent times. Polyethersulphone (PESP) ultrafiltration membrane was also examined for the MP removal capacity, and this membrane was able to remove almost 91–96% of nano sized PE, PVC and PES from the aqueous media.^42^ Hydrophovic Polyvinylidene Fluoride (PVDF) microfiltration and ultrafiltration membrane having pore size of 0.22 µm which showed the efficiency of complete removal of PP and PVC MPs under controlled laboratory condition.^43^ Previously developed PVDF ultrafiltration membrane showed the efficiency of about 85–90% only.^42^ Polypropylene (PP) – hollow fiber (HF) microfiltration membrane, which could remove MPs mixture of PE, PP, PET, PS, Nylon, and PVC from synthetic sea water prepared in the laboratory with a high efficiency of about 99%.^43^ Researchers are working on the development of more advanced membrane by using emerging material. A novel membrane was prepared by mixing ZIF-8 with wood aerogel and was used for the removal of PVDF and PS which showed removal efficiency as high as 91% and 85% respectively.^44^ A microporous membrane was also prepared from poly (piperazine-amide) coated silica based ceramic hollow fiber which was able to effective remove micro sized particles of g PAN, PVC, PVP, and PMMA.^45^ Another porous membrane was developed from organic polymeric substance g 1,4-phenyldimercaptans and 1,3,5-triacetylbenzene which showed the efficiency of 90% removal of PS MPs from aqueous media.^46^ The remaining microplastic in liquid sample are generally remove through further treatment such as oxidation, coagulation–flocculation, as well as sedimentation. So, proper management of these microplastics remains an important challenge in this remediation method. Besides having high removal efficiency this particular process has some disadvantages also. Although it is a dynamic process, the main issue is the membrane contamination which leads to a decreased filtration rate.^47–49^ Due to continuous filtering of contaminants, the pores of the membrane get blocked, and contaminant cake is formed on the membrane surface which ultimately results into the membrane fouling and decrease in the flux of filtrate.^50^ The production cost of such advanced membrane is too high and the requirement of frequent changes of the membrane due to the clogging effect adds additional cost to it which makes the process a bit non-feasible. The treatment and management of concentrated by-products remain a major challenge in membrane-based remediation method. No such membrane is still developed which can effectively remove nano-scale particles.

### 4.2. Density separation

**Fig. 4:**
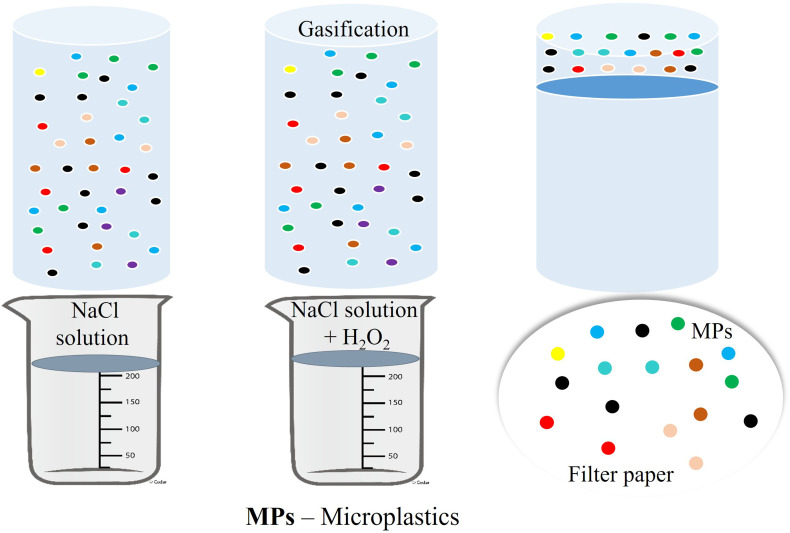
Schematic representation of density separation and oxidative digestion process for extraction of microplastics using NaCl solution and H_2_O_2_.^9,51^

It is a very commonly used process for the separation of MPs from different environmental media. The main principle lies on the flotation of light weight MP particles on the upper layer of the solution having a greater density than that of the MPs which is shown in Fig. 4.^9,51^ Different kinds of concentrated salt solutions like sodium chloride, sodium bromide, sodium iodide, zinc chloride, calcium chloride *etc.* are generally used as the medium for the density separation of MPs.^52,53^ A new method for density separation process by applying moderate heat to monosodium phosphate (NaH_2_PO_4_) solution which increased its density making the process more efficient.^52^ A process was also developed for the recycling of sodium iodide after repeated use in the density separation of MPs without any sign of changes in the density through proper filtration and distillation so that a single solution can be used numerous times to cut the cost of this method.^54^ To separate MPs from solution by using saturated NaCl solution by mixing it slowly with air flux but the process didn't work for high density poly ethylene like PET.^55^ The density separation process is still not used for the removal of MPs but only used for the analysis purpose because of the lack of appropriate operating protocol, difficulties in the extraction of the high concentration salt solution from the natural media after the separation of MPs which can create toxicity among the organisms and also some salt which show high efficiency in removing MPs are expensive.^9,56^

### 4.3. Adsorption

**Fig. 5:**
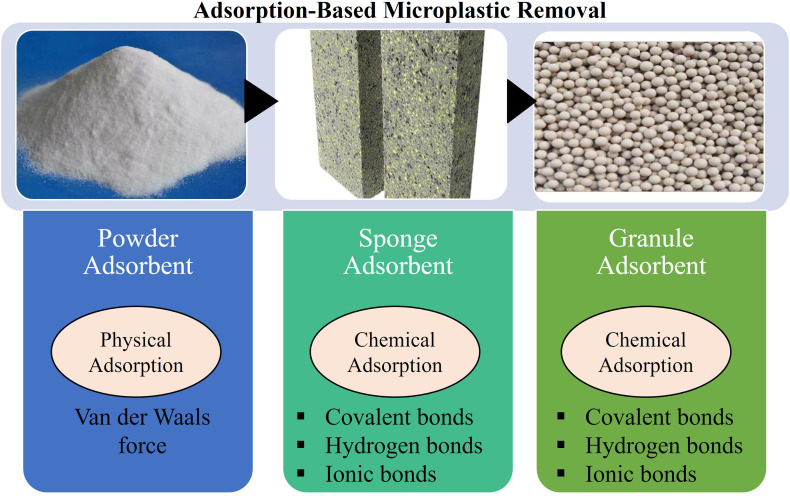
Different types of adsorbent (Powder Adsorbent, Sponge Adsorbent, and Granule Adsorbent) materials used for microplastic remediation.^57,58^

Adsorption is a very conventional method used for the treatment of wastewater for the removal of a variety of contaminants and recently in case of MP removal this process is being experimented. In Fig. 5. Shown that there are two types of adsorptions, chemical and physical adsorption. Chemical adsorption is a very selective process and refers to the destruction and formation of inter-molecular chemical bonds like covalent bonds, hydrogen bonds and ionic bonds *etc.* of the adsorbents and the adsorbate which generally requires elevated temperature.^57^ On the other hand, physical adsorption is non-selective compared to the chemical adsorption and based on the intermolecular forces or van der Wals force which can be operated in normal temperature with high efficiency.^58^ The adsorption efficiency greatly depends on the shape and size of the MPs. For instance, the adsorption rate of the irregular shaped MPs are higher than the smooth spherical micro beads.^35^ In case of MP adsorption, the adsorbate can be used in two forms for their different operating processes, powder and sponge. The powder adsorbents are first mixed with the solution containing MPs and then the adsorbents along with the MPs are separated from the solution by different physical processes like centrifugation, filtration or magnetic separation.^35,59^ Fly ash was modified by mixing with iron ions which evolved with high surface function ability and magnetic property for the successful removal of PS particles after mixing and centrifuging.^59^ In case of sponge adsorbent, the porous structure of the sponge can efficiently absorb the MP particles and separate them from solution. A robust and compressive sponge combining chitin and graphene oxide which showed excellent efficiency for the removal of MP particles and beads.^60^ These chitins based adsorbent sponges were also biodegradable in nature and can be used multiple times after washing. Granular adsorbents are also being used for removing MPs from solutions which can be synthesized commercially for the treatment of MP contaminated water. A unique Zn–Al layered double hydroxide granule was prepared which gained high success in removing even nano-sized plastic particles with an efficiency of almost 96%.^61^

Polystyrene nanoplastics have been effectively removed using by activated carbon and biochar adsorption systems because their high surface chemical energy increases attachment to adsorbent surfaces.^62^ Similarly, aluminium-based coagulants promote aggregation of PE and PS nanoplastics through electrostatic interactions and surface PE and PS nanoplastics through electrostatic interactions and surface charge neutralization.^63^ Electrocoagulation also facilitates nanoplastic removal by generating metal hydroxide flocs that adsorb suspended nanoparticles and improve their separation from wastewater treatment plant.^64^ The high surface chemical energy of nanoplastics promotes strong interactions with pollutants, dissolved organic matter, and ions in the aquatic system, leading to stable colloidal suspensions that hinder aggregation and separation. In addition, the adsorption of contaminants can alter the nanoplastic surface properties and reduce treatment efficiency. High surface reactivity may also increase membrane fouling and reduce the treatment efficiency during filtration and adsorption processes Consequently, conventional remediation methods often show limited effectiveness, while advanced treatment systems require higher energy input, operational complexity, and treatment cost for efficient nanoplastic removal. In the case of natural adsorbents, the operation process is very slow, and the adsorption capacity is also not that satisfactory compared to the synthesized adsorbents. The adsorbents cannot be reused frequently which creates huge waste problem. The synthesized adsorbents are more effective in removing MPs but the synthesis processes as well as the raw materials used for the commercialization of the adsorbents are much more expensive that the process is not economically beneficial most of the time to be used in large scale. Membrane-based adsorption treatment currently represents the most efficient physical approaches for rapid microplastic removal, although highly cost, and fouling remain major barriers for large-scale application.

## 5. Chemical process for the removal of microplastics

### 5.1. Coagulation–flocculation

It is one of the most conventional processes for the removal of suspended solids in the wastewater treatment process. Mainly hydrophobic substances can be effectively removed from the solution by this process. The MP particles are negatively charged colloidal particles which remain suspended in the aqueous media. The addition of chemical coagulants neutralizes the charge of the MPs and helps them to aggregate and large flocks are formed which can be easily separated as they precipitate to the bottom of the solution due to increased weight and density.^65^ Larger particles are then removed by some secondary treatment like filtration, sedimentation *etc.*^66^ Most used coagulants for the MP removal are aluminum chloride and iron chloride but the removal efficiency is not that much satisfactory. Al-based coagulant, Fe-based coagulant and some synthetic organic material-based coagulant were experimented thoroughly for the removal of MPs.^53^ Addition of some polymeric substances like polyacrylamide and polymer coated sand along with the coagulant can increase the formation of large flocks of MPs to be removed from the system.^9^ The effectiveness of this process largely depends on the MP properties such as mould into flocks as they are not easily attach to each other compared to those which are larger and have irregular shape.^67^ In such cases secondary treatment is needed for the complete removal of MPs from water like in the experiment of coagulation was followed by ultra-filtration while removing micro sized particles of PE from aqueous media by using aluminum chloride and iron chloride as coagulant.^66^ From their observation it was stated that smaller size MPs removal rate became higher after the addition of a large amount of AlCl_3_. However, the removal efficiency gets affected by the presence of organic matter in the solution, the ionic strength of the solution and turbidity while it also depends on the pH of the solution.^36^ The major advantage of this process is it requires the addition of a huge amount of chemical coagulant which is not environmentally safe. The presence of Al-salt in the drinking water which remains as a residue of added coagulant has tremendous adverse effects on the living beings.^68^

Besides the development of removal technologies, several regulations and strategies are being implemented to overcome this serious environmental issue throughout the globe. The extensive plastic strategies are undertaken by the European Union to stop the release of MP particles into water body so that waterborne microplastic contamination can be reduced.^69^ Besides, strategies like reducing the production and discharge of MPs directly at the source, recycling of the already produced MPs and promoting the use of bio-based products should be adopted to ameliorate the harmful effects of MPs on the total environment.

Brownian motion is referred to as the random and continuous movement of nano-sized particles dispersed in a fluid due to constant collisions with surrounding molecules of the medium.^70^ Advanced treatment methods such as coagulation, membrane filtration, and electrocoagulation have shown effective removal of nanoplastics from wastewater treatment plants. These techniques promote physical retention of nano-sized particles. These advanced treatment methods are considered more effective than conventional sedimentation techniques because they can overcome the strong dispersion behaviour caused by Brownian motion.^63^ It has significant challenges for nanoplastic removal. Their extremely small size and high mobility reduce sedimentation and filtration efficiency, while their high surface chemical energy promotes interactions with pollutants and organic matter. As a result, conventional treatment methods often show limited effectiveness, and advanced multi-stage remediation methods with higher energy and effective requirements are generally needed for efficient nanoplastic removal.

### 5.2. Photo-catalysis

**Fig. 6:**
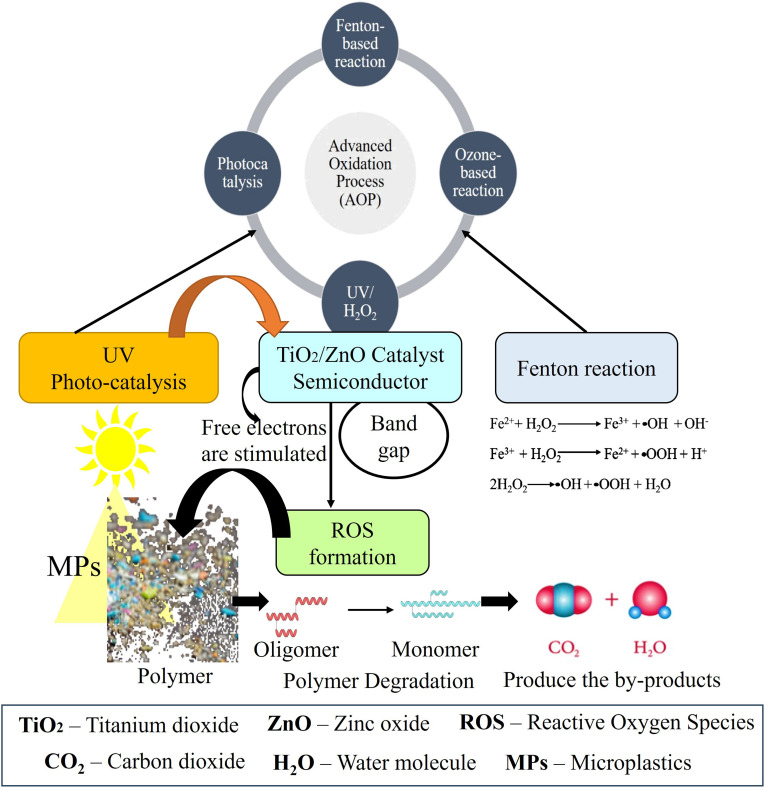
Photocatalytic mechanisms of microplastic degradation under UV irradiation using TiO_2_/ZnO catalyst.^72,73^

The photo-catalysis process and Fenton treatment are an Advanced Oxidation Process (AOP) that degrades the organic and inorganic pollutants using light-activated pollutants.^71^ In Photo-catalysis process (Fig. 6), nanoscale metal oxide and metal oxide compound semiconductors such as TiO_2_, ZnO, Fe_2_O_3_, ZnS, and CdS are generally used for the degradation of microplastics,^72^ and Fenton reaction occurs in acidic media and produces hydroxyl radical (OH^−^) when hydrogen peroxide (H_2_O_2_) reacts with ferrous ions (Fe^2+^).^73^ TiO_2_, and ZnO semiconductors are most often used for the presence of appropriate band gaps rather than others. When they interact with UV light, as a result, free electrons are stimulated by light with energy higher than the band gap, these excited electrons shift from the valence band to the conduction band and produce the reactive oxygen species including hydroxyl (OH˙) and superoxide (O^2−^) radicals. Finally, ROS reacts with MPs resulting in the splitting, cross linking, and breakage of the long polymeric chains. These chemical changes show discoloration, corrosion, cracking, and erosion on the plastic surface. The degradation process continues when the ROS reacts with deeper polymer layers and produce intermediate products such as alcohols, carboxylic acid, aldehyde. as well as ketones and finally produces CO_2_ and H_2_O as by-products after full breakdown of the long polymeric chain.^70^ The toxicity of these intermediate products are impact on the ecosystem, health risk. These intermediate products can exhibit cytotoxicity, oxidative stress, and adverse effects on aquatic organisms as well as human health.^71^ So, incomplete photocatalytic degradation may lead to secondary environmental risks, emphasizing the need for efficient mineralization and careful monitoring of degradation by-products during remediation processes. Several studies have been carried out on the mechanism and degradation potential for TiO_2_^−^ based nano devices and micro-motors in MP photo-catalysis. Using Au-decorated TiO_2_^−^ micro motors, MPs have been treated in the photo-catalytic process to increase effectiveness.^76^ Since this MP treatment method is relatively new compared to other conventional and advanced treatment approaches, further study is necessary to develop more effective and efficient advanced photo-catalysts that can effectively be used for the removal of MPs in wastewater. Also, Fenton treatment has been shown in several experiments to effectively break down plastic waste into other by products.^77^ When UV light acts together with Fenton reaction it is referred to as Photo-Fenton process. An electron shifts from an orbital centered on the binding agent to another orbital centered on the metal when Fe^3+^ aquo-complexes are exposed to radiation. This transference, which is known as ligand metal charge, indicates the conversion of Fe^3+^ to Fe^2+^ and that the ligand has been oxidized, resulting in the production of OH radicals. For the Fenton reaction, a pH value between 2.5 to 3.0 is ideal. Above pH 3, iron begins to precipitate as hydroxides.^78^ The efficiency of Fenton treatment depends on the chemical composition of plastic polymer. PE, PP, and PS are more susceptible to disintegration for the presence of simpler carbon bonds.^79^ PET and polymethyl methacrylate (PMMA) break down during Fenton treatment to the presence of ester bonds.^80^ However, PVC is less affected because its chlorine component interferes with the radical reactions.^81^ Photocatalytic degradation of PE using TiO_2_ photo catalyst along with nearly 16.98% mass loss after 12 hours of UV treatment, indicating partial oxidative degradation of the PE matrix rather than complete mineralization. It was reported that produce oxygen containing in intermediate functional groups such as aldehyde and ketones, with a significant an increase in the carbonyl index from 0.189 to 0.1350, confirming oxidative chain scission and surface modification of the PE polymer during UV irradiation.^82^ Photocatalytic degradation of PE microplastics using ZnO photo catalyst, observed 30% increase in the carbonyl index, indicating the formation of intermediate products such as aldehyde and ketones during oxidation.^83^ PE, and PP microplastics reported the formation up to 18 wt% low-molecular-weight oxidation processes.^84^ These degradation intermediates increase environmental toxicity and pose risk to aquatic organisms and human health due to their higher mobility and bioavailability. Chemical remediation methods provide effective degradation of persistent MPs, however, concerns regarding energy demand and secondary pollutant produce further optimization for sustainable implementation.^74,75^

## 6. Biological process for the removal of microplastics

These so-called conventional techniques, both physical and chemical, for the removal microplastics frequently fall short of eliminating these particles entirely or necessitate a large investment of infrastructure and energy.

Biological degradation has become a viable, environmentally benign strategy to reduce microplastic contamination in recent years. This process breaks down plastic polymers into simpler, non-toxic molecules by using living creatures like bacteria, fungi, algae, and enzymes. It has been shown that some microbes can grow on plastic surfaces, create biofilms, and release enzymes that can break down manmade polymers. For example, several species have demonstrated differing degrees of success in breaking down polyethylene, polystyrene, and polyethylene terephthalate (PET), including *Pseudomonas*, *Ideonella sakaiensis*, and *Aspergillus niger*. Table 1 summarize the use of different types of biological agents, and their enzymes degrade or remove the different polymers under different environmental and different time conditions. For the degradation and removal of plastic particles in different times. In the environment, when microorganisms break down the chemical structure of any substances from highly complex to less complex molecules through enzymatic activities or metabolic pathways, the process is known as biodegradation.^85^ The degradation of microplastic by living organisms depends on different factors, including types, shapes, size, chemical composition of the polymer, and diverse environmental conditions. There are several steps involved in the degradation of MP by bio-organisms (Fig. 2). At the very primary stage, microbial cells start colonizing on the surface of the plastic particle where they secret the Extracellular Polymeric substances (EPS) to form biofilm over the surface. These enzymes help to break down the long chain of the targeted polymer and convert it into oligomer and monomer. Finally, through the mineralization process, intracellular enzymes convert these into CO_2,_ H_2_O, biomass, energy in aerobic condition, and CH_4_, CO_2,_ biomass, energy in anaerobic condition.^86^

**Table 1:** A comprehensive comparison of physical and chemical treatments of MPs, with specific values for specific types of microplastics

Remediation method	Categories of remediation method	Polymer types	Condition	Degradation efficiency	Source	Advantages	Limitations	Ref.
Membrane filtration	Polyethersulphone (PESP) ultrafiltration membrane	PE, PVC and PES	Aqueous media	91–96%	Wastewater treatment plants	High efficiency	High cost	42
	Hydrophobic polyvinylidene fluoride (PVDF) microfiltration and ultrafiltration membrane	PP and PVC	Laboratory condition	85–90%	Wastewater treatment plants	High efficiency	High cost	42
	Polypropylene (PP) – hollow fiber (HF) microfiltration membrane	PE, PP, PET, PS, Nylon, and PVC	Laboratory condition	99%	Sea water	High efficiency	High cost	43
Adsorption (Zn–Al layered double hydroxide granule)	—	Nano-scale plastic debris (NPDs)	Aqueous media	96%	—	High efficiency	High cost	61
Photocatalytic degradation TiO_2_ photo catalyst	—	PE	Laboratory condition	16.98% mass loss	—	High efficiency	High cost	82

Activated sludge systems, membrane bioreactors, and biofilm reactors are widely investigated for the efficient removal of MPs through a combination of biological and physical treatment. Hydraulic retention rate (HRT), aeration rate, oxygen transfer efficiency are play a major role between MPs and different microorganisms. Continuous flow reactor systems are particularly important for wastewater treatment applications because they enable large scale and continuous treatment of MP contaminated water under effective condition. Pilot-scale wastewater treatment studies, kinetics analysis, and mass transfer methods provide important engineering studies, regarding the feasibility, scalability, and operational optimization of biological MP remediation technologies for real world environmental applications.

### 6.1. Phytoremediation of microplastic

Phytoremediation is the process of using plants to help remove pollutants from the environment. The concept of using woody plants for remediation of the MPs has been promoted by the use of the roots of birch trees.^87^ Barley is used in a variety of hydroponic systems for the phytoremediation of microfibers, to improve bio-technologies for the removal of MPs.^88^ Developing successful phytoremediation techniques, understanding the potential risks of MP pollution, and assessing the possible effects on human and animal health should be the main goals of future research.^89^ A different approach claiming direction for future study is the role of plant–microbe interactions in improving phytoremediation accuracy for soil contamination with heavy metals and MP.^90^ Natural gas, oil, coal, and their byproducts are common sources of plastic, a synthetic polymeric material comprising many components, including carbon, hydrogen, oxygen, nitrogen, silicon, and chlorine.^91^ MPs get broken down by oxidation and biological processes. The enzymatic activity of microorganisms causes biological degradation, which disrupts the structure of the polymer. Light exposure and reactive oxygen species are involved in oxidation processes, which include photochemical oxidation, and photo degradation. Biological degradation is also influenced by pH, moisture content, and temperature.^92^ Spirodelapolyrhiza is a free-floating plant that has roots, streams, and leaves that are readily coated in MPs.^93^

### 6.2. Bacterial degradation

Bacteria are prokaryotic cells that have DNA in the nucleoid region. The simple and tiny structure of bacteria, typically ranging from 1 to 10 micron, is only 1/10 to 1/100 000 of that of eukaryotic cells. Its basic binary division mechanism generates a faster rate of replication compared to other species. Biofilm formation is also necessary for the bacterial degradation of MPs because it makes it simpler for bacteria colonies to adhere to MP surfaces and increases their lifespan.^94^*Bacillus* and *Rhodococcus* species from mangrove sediments are degrading the PP-type MPs. For PP, the degradation efficiency of *Bacillus* and *Rhodococcus* species is 4.0% and 6.4%, respectively. Two bacterial strains, Bacillus cereus and *Bacillus gottheilii* are identified in a related study and are found to break down different types of MPs from mangrove sediments.^94^ The degradation efficiency of B. gotthiili for PS, PET, and PE is 7.4%, 6.6%, and 1.6%, respectively.^89^ Microorganisms break down MPs through a variety of biological processes. Several kinds of enzymes, including lipase, protease, glycoside hydrolases, laccase, urease, and esterase, are secreted from some bacteria. Which include *Bacillus* species, *Ideonellasakaiensis*, *Rhodococcus* species, and *Paenibacillus* species. These enzymes attach to the backbone of long-chain plastic polymers and break them down into monomer units. The initial and crucial step in the degradation of microplastics is enzymatic hydrolysis, which enhances their hydrophilicity by targeting the functional groups of MP polymers.^95^ Various Gram-positive bacteria, including Staphylococcus epidermidis, and Enterococci sp. as well as Gram-negative bacteria, including *Vibrio chloerae*, *Pseudomonas fluoresces*, *Pseudomonas aeruginosa*, and *Escherichia coli*, have been shown to produce biofilms. By increasing their resistance to antibacterial agents, bacteria biofilms show sophisticated growth through gene regulation. Microorganisms form unique habitats within their biofilms and can survive challenging conditions like Osmotic stress, dehydration, UV radiation, and pH changes.^96^*Ideonella sakaiensis* isolated from soil and produce two types of enzymes, PETase and MHETase completely degrade the polymer chains of PET which is commonly found in packaging and plastic bottles under aerobic conditions at 30 °C and 300 strokes per min within 42 days.^97^

PETase breaks the ester and produces two intermediate products, which are ethylene glycol and terephthalic acid that are further metabolized by bacteria and produce non-toxic compounds.^98^ Klebsiella sp. EMBL-1isolated from larval and produce enolase, aldehydye dehydrogenase, catalase-peroxidase, and oxygenase degraded the PVC, resulting in 19.57% weight loss after 90 days at 30 °C under aerobic shaking conditions.^99^*Acinetobacter venetianus* F1 obtained from cold seep sediments showed 12.2% degradation of PE through the activities of hydrolases, reductases, esterase, lipase, oxygenase, and dehydrogenase after 56 days at 28 °C.^100^ Other bacteria strain such as *Bacillus* sp., *Pseudomonas* sp., *Stenotrophomonas Enterococus*, *Acinetobacter Bacillus* sp., *Paenibacillus* sp., *Beta protebacteria*, *Rhodococcus jostii*, *Rhodanobacter* sp., *Stenotrophomonas maltophilia*, *Enterobacter asburiae*, *Chelatococcus*, *Pseudomonas*, and *Paenibacillus amylolyticus* has ability to degrade polymer such as HDPE, PVC, PS, PE, and PLA under aerobic conditions with varying degradation efficiencies and exposure durations.

### 6.3. Fungal degradation

Microplastics are broken down by fungi through the mechanism of physical colonization, biochemical reactions, and enzymeatic activity. *Aspergillus*, *Penicillium*, and *Cladosorprium* are the primary fungal species that break down polyethylene (PE). These microorganisms use PE as a primary carbon source and break down the polymer with the help of extracellular enzymes. These fungi reduce hydrophobicity and break down the chemical bonds of plastic substances.^101^ When fungi affect the plastic surface, the physical characteristics of plastic, such as molecular weight, rate of fragmentation, and yield strength, are changed, as well as the chemical properties, including functional groups, are also changed.^102^ Based on their morphological characteristics, fungi are classified as dimorphic, filamentous, or yeast-like eukaryotic organisms. Filamentous fungi, including *Fusariumfalciforme*, *Aspergillus* species, *Purpureocillumlilacinum*, and *Fusariumoxysporum*, are important to the mineralization of micropollutants in the environment.^103^ Aspergilus tubingensis isolated from soil and 90% degrade the polyurethane (PUR) through esterase and lipase activities at 37 °C under aerobic shaking conditions within 60 days.^104^*Phanerocheate chrysosporium* degraded 31% of PVC using lignin peroxidase under acidic aerobic conditions.^105^ Other fungal species including *Alternaria alternate*, *Chaetomium globosum*, *Monascusruber*, *Pestalotiopsis microspore*, *Aspergillus oryzaes*, *Aspergillus niger*, and *Aspergillus* have been reported to degrade polymers such as PE, PVC, PET, HDPE, and PUR under different environmental conditions, through oxidoreductase, laccase, lipase, esterase, and peroxidase enzyme activities. Fungal-mediated breakdowns of microplastics potentially occurs in different stages, these are biodeterioration, biofragmentation, and mineralization. The biodeterioration process is an initial stage where their physical colonization and enzyme activity degrade the plastic polymers. Extracellular enzymes like peroxidase, laccase, and esterase are released by these microorganisms and break down the PE, PP, and PET polymer chains. These enzymes stimulate hydrolytic and oxidative reactions that help break down microplastics into smaller molecules. Filamentous fungi produce hydrophobin proteins by their gene expression, enzyme secretion and self-assembly. These proteins make amphipathic monolayers at the interface between hydrophobic and hydrophilic substances. These surface-active proteins contain 70–350 amino acids and are involved in the formation of hyphae. The hyphae release of extracellular enzymes leads to minimize the frictional properties of different plastic polymers. Several factors, such as different polymer types, environmental factors including biotic and abiotic conditions, and plastic composition influence the rate of fungal degradation. It is crucial to understand the mechanisms and efficiency of fungus-driven MP remediation to develop sustainable and environmentally suitable techniques to solve MP contamination. Extracellular and intracellular enzymes convert polymers into monomers. Through the mineralization process, methane is produced under anaerobic conditions as well as carbon dioxide and water are produced under aerobic conditions.

### 6.4. Microalgae degradation

Algae can remove microplastics through interception, capture, and entanglement while also promoting their aggregation through extracellular polymeric substances (EPS). The secretion of EPS interaction between microalgae and microplastics leads to heterogeneous aggregation.^106^ MPs are effluent from wastewater at an excessive rate by the existing wastewater treatment plant. Consequently, novel processes are invented to fully and successfully remove MPs to mitigate the risk to the aquatic environment. The recent studies, algae do exceptionally well in the cleanup of MPs.^107^ Based on the research *Chaetocerosneoracile* and *Rhodomonassalina* fix with MPs in the marine ecosystem from the ocean floor to deper layers through phytoplankton aggregates.^108^ and rapidly removes potentially very high amounts of plastic.^108^ In the study area Great Lakes region, the *Cladophora fresh* filamentous green algae capture the MPs by physical entanglement and adsorption process.^109^ Large algae can capture and hold into MPs in seawater through entanglement, adhesion, encapsulation, embedding, and epidermal biological capture. *Scenedesmus abundans* can effectively remove greater than 84% from water bodies under laboratory condition by heterogeneous aggregation of polystyrene (PS), poly methyl methacrylate (PMMA), and polylactide (PLA) MPs.^110,111^ Here initial MP concentration range of 10–100 mg L^−1^ with an exposure time of more than 2 days. The algae *Gloeocapsa* sp. produces a significant amount like 2.146 mg Ml^−1^ Extracellular Polymeric Substances (EPS) and shows MP aggregation capability. Also freshwater algae *Cyanothece* sp. has been found to produce EPS with strong biofloculant properties.

**Fig. 7:**
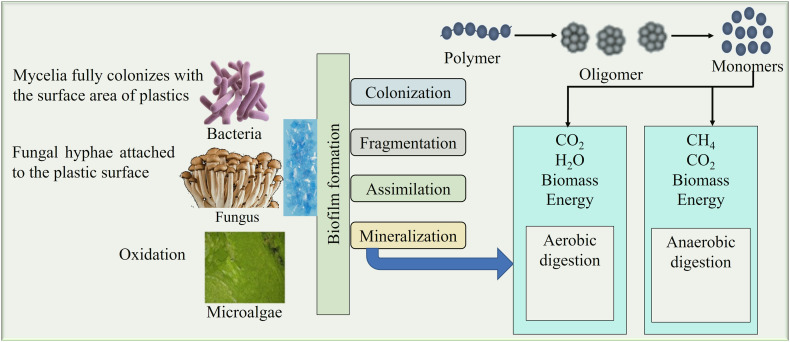
The mechanism of bio-disintegration of plastic particles and the production of organic and inorganic substances in aerobic and anaerobic conditions.^86^

*Phormidium lucidum* isolated from domestic sewage degraded approximately 30% LDPE through laccase and peroxidase production under aerobic conditions within 42 days.^112^*Scenedesmus dimorphus*, *Navicula pupula*, and *Anabaena spiroides*, are degraded different degrres of LDPE. *Phaeodactylumtri cornutum*, *Spirulina* sp., *Diatoms*, and *Chlamydomonas reinhardtii* have ability of degradation odF PET and PP through PETase and extracellular enzyme-mediated mechanisms. The interaction between MPs and algae highlights the potential of microalgae-derived biopolymers as sustainable and environmentally eco-friendly substitutes for synthetic flocculants in WWT plants. Various microalgae have shown potential in MP remediation, such as *Tetraselmis* sp. has shown a significant ability to remove the MPs, but it depends on the size of the MP particles. In Fig. 7 shown the process of bio disintegration of tiny plastic particles. Biological remediation offers an eco-friendly and sustainable strategy for microplastic degradation, but degradation rate, degradation process and scale-up feasibility are necessary for real world environmental applications. In Table 2 discussed about degradation of different types MPs by bio-organisms such as bacteria, fungi, and microalgae.

**Table 2:** Degradation of different types MPs by bacteria, fungi, and microalgae

Types of micro-organisms	Name	Source	Types of plastic	Enzymes	Degradation condition	Plastic weight loss	Time	Ref.
Bacteria	*Ideonella sakaiensis*	Soil	PET	PETase, MHETase	30 °C	100%	42 days	97
					Aerobic			
					300 strokes per min			
	*Klebsiella* sp*.* EMBL-1	Larval gut	PVC	Enolase, aldehyde dehydrogenase, catalase-peroxidase, oxygenase	30 °C	19.57%	90 days	99
					Aerobic			
					150 rpm			
	*Acinetobacter venetianus* F1	Sediments from the Haima cold seeps	PE	Hydrolases, reductases, esterase, lipase, oxygenase, dehydrogenase	28 °C	12.2%	56 days	90
					160 rpm min^−1^			
					Aerobic			
	*Bacillus* sp.	Discarded refuse	HDPE	—	30 °C, aerobic	23.14%	4 weeks	128
	*Pseudomonas* sp.	Larval gut	PVC	—	30 °C	6.13%	30 days	129
	*Stenotrophomonas*				180 rpm min^−1^			
	*Enterococus*				Aerobic			
	*Acinetobacter*	Landfill	PE	—	30 °C, aerobic	14.7%	60 days	130
	*Bacillus* sp.							
	*Paenibacillus* sp.							
	*Beta protebacteria*	Laboratory	PET	—	30 °C	31.2%	60 days	131
	*Rhodococcus jostii*				Aerobic			
	*Rhodanobacter* sp.	Soil	PE	—	28 °C	—	—	132
					180 rpm min^−1^			
					Aerobic			
	*Stenotrophomonas maltophilia*	River sediment and soil	PS	—	30 °C	43.5%	60 days	133
					110 rpm min^−1^			
					Aerobic			
	*Enterobacter asburiae*	Plastic-eating	PE	—	—	6.1 ± 0.3%	28 days	134
		Waxworms						
	*Chelatococcus*	Compost	PE	—	—	—	80 days	135
	*Pseudomonas*	Digester sludge	PLA	—	—	—	40 days	136
	*Paenibacillus amylolyticus*	Soil samples	PLA	Protease	—	—	14 days	137
				Esterase				
Fungi	*Aspergillus tubingensis*	Soil	PUR	Esterase, lipase	37 °C	90%	60 days	138
					150 rpm min^−1^			
					Aerobic			
	*Phanerocheate chrysosporium*	Laboratory	PVC	Lignin peroxidase	25 °C pH = 5	31%	28 days	139
					Aerobic			
	*Alternaria alternata*	Plastic waste from coastal region	PE	Peroxidase, laccase. Oxidoreductase	25 °C	—	—	31
					Alkali, aerobic			
	*Chaetomium globosum*	—	PVC	—	—	—	After 28 days	140
	*Monascusruber*	—	PPU	—	—	—	After 14 days	141
	*Pestalotiopsis microspora*	—	PPU	—	—	—	After 16 days	142
	*Aspergillus oryzaes*	—	PET	—	Temp 30 °C	26%	—	143
	*Aspergillus niger*	—	HDPE	—	30 °C	3.44%	—	144
	*Aspergillus cremeus*	—	HDPE	—	37 °C	8%	—	145
Microalgae	*Phormidium lucidum*	Domestic sewage	LDPE	Laccase, peroxidase	Aerobic	30%	42 days	146
	*Scenedesmus dimorphus*	—	LDPE	—	—	3.74%	—	147
	*Navicula pupula*	—	LDPE	—	—	4.44%	—	147
	*Anabaena spiroides*, *Cyanobacterium* (blue-green alga)	—	LDPE	—	—	(8.18%)	—	147
	*Phaeodactylumtri cornutum*	—	PET PETG	PETase	—	—	—	148
	*Spirulina* sp.	—	PET, PP	Extracellular enzymes	—	—	—	149
	*Diatoms* (in mesophilic conditions)	—	PET	PETase	—	—	—	150
	*Chlamydomonas reinhardtii*	—	PET	PETase	—	—	—	151

### 6.5. Enzymatic action

**Fig. 8:**
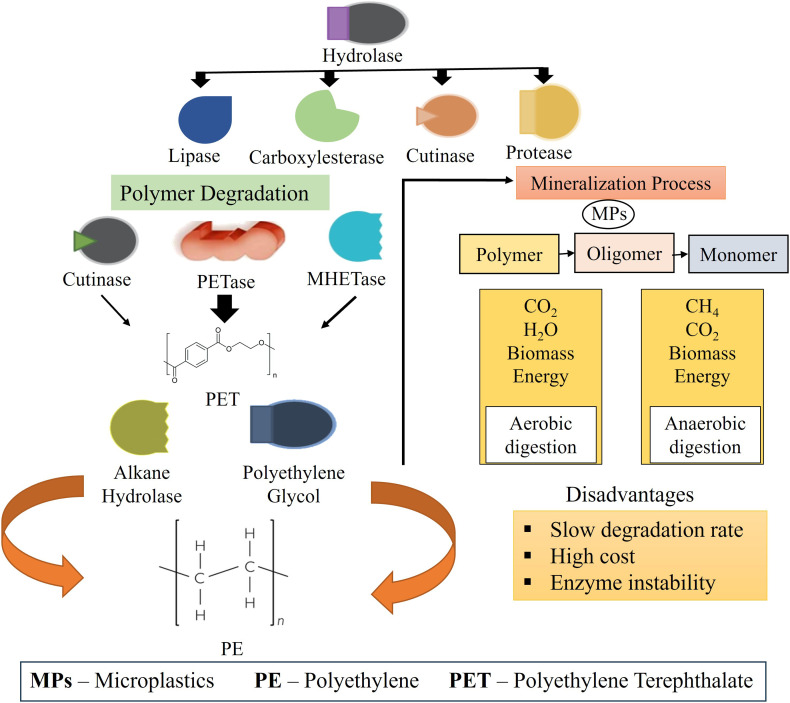
Degradation of polymer using different enzymes and disadvantages of enzymatic reaction for removal the MPs.^29^

The sustainable and eco-friendly process of microbial degradation of MPs breaks down plastics into monomers, oligomers, CO_2_, and H_2_O through enzymes generated by microorganisms,^113^ this mechanism is known as depolymerization and mineralization mechanism which is shown in Fig. 8.^29^ Another Enzyme Surface Modification Mechanism involves mainly hydrolase enzymes *e.g.* lipase, carboxylesterase, cutinases, and proteases which can degrade the MPs and increase their susceptibility.^114^ Elemental Spectroscopy Chemical Analysis (ESCA) is used to identify and explain the mechanism of interaction between the enzyme and the surface region of MPs. Different types of plastic polymers are present in nature, and each one can be broken down by different types of enzymes which depend on different metabolic processes. These enzymes include amidase, cutinases, carboxylesterases, esterases, hydrolases, and lipases.^115^ The two categories of enzymatic breakdown of MPs are hydrolyzable (PET) and non-hydrolyzable (PE, PS, and PP). Cutinase, PETase, and MHETase enzymes are reported for breaking down PET secretes the enzyme cutinase, which breaks down the polyester's aliphatic and aromatic ester linkages through the breakdown of PET.^116^ PETase enzyme breaks down PET by hydrolysing the ester group (–COO–). PET consists of ethylene glycol and terephthalic acid connected through ester linkages.^117^ This enzyme reacts with the PET surface and activates a H_2_O molecule, which attacks the carbonyl carbon of the ester bond. Through this reaction, break down the polymer chain and produce intermediate products such as Mono (2-hydroxyethyl) terephthalate (MHET), bis (2-hydroxyethyl) terephthalate (BHET), and terephthalic acid (TPA).^118^ The MHETase enzyme specifically targets MHET, which is an intermediate product produced during PET breakdown. This enzyme hydrolyses the ester bond in MHET and converts it into TPA and EG.^119^ This reaction completes the breakdown of PET into its monomers. Cutinase enzyme breaks ester bonds in both natural and synthetic PET. It binds to PET and hydrolyses the ester linkages in the polymer chain, and produces oligomers such as BHET and MHET, which are further converted into TPA.^120^ These three enzymes cannot break down PE, PP, and PS because these plastics do not contain ester bonds. They consist mainly of non-polar C–C and C–H bonds, which resist enzymatic hydrolysis. PE polymer chain degrades by laccase, manganese peroxide (MnP), lignin peroxidase (LiP), and alkane hydroxylase (AlkB system).^121,122^ These enzymes primarily target the inert PE surface and bring oxygen into the C–H bonds of the PE polymer chain. As a result, alcohols, carbonyls, and carboxylic acids are produced.^123^ This reaction reduces the hydrophobicity and stability of the polymer. PP polymer chain degrades by alkane monooxygenase, cytochrome P450 monooxygenase, laccase, manganese peroxide (MnP), and lignin peroxidase (LiP).^124^ These enzymes specifically target the more reactive tertiary carbon atoms in PP. This leads to the formation of unstable hydroperoxide, which subsequently decomposes into carbonyl-containing compounds. These oxidative reactions weaken the polymer chain and promote random chain scission, producing smaller oxidative fragments that can be assimilated and further degraded by microbial metabolic pathways. PS polymer chain degrades by styrene monooxygenase, styrene oxide isomerase, laccase, and manganese peroxide (MnP), and lignin peroxidase (LiP) enzyme.^125^ These are converted styrene to styrene oxide, further these enzymes are modified the aromatic ring, and produces some intermediate compounds such as phenyl acetaldehyde and catechol derivatives. PS breakdown depends mainly on oxidation-driven aromatic ring modification followed by microbial assimilation. Microorganisms Ideonellasiakensis secretes the PETase enzyme, which breaks polyester at pH values 7 to 9 by breaking its aromatic ester link.^126^ This enzyme disrupts amino acids, which prevents the thermal equilibrium from forming. MHETase attacks polyester's carbon atom nucleophilically. It needs a temperature of 45 °C and an ideal pH of 6.5 to 9.^126^ Whereas two intracellular enzymes, such as alkane hydrolase and polyethylene glycol (PEG) dehydrogenase, completed the breakdown of PE. At an optimal temperature of 45 degrees C and pH 4.5, alkane hydrolases break polyethylene whereas PEG generates glyoxylic acid.^127^

## 7. Bioremediation in comparison with physical and chemical treatment

Bioremediation is a sustainable and eco-friendly treatment for environmental management. This method is efficient, economical, and repeatable. Physical, chemical, and biological remediation of MPs. have some advantages and disadvantages which is mentioned in Table 3. Physical treatments, including membrane filtration, density separation, and adsorption, are commonly used because of their efficient separation capability and relatively simple technique. Chemical treatment such as coagulation–flocculation, and photo catalysis enhance microplastic aggregation and degradation, but their energy demand is high and produce secondary pollutants. Biological remediation approaches are regarded as sustainable and ecofriendly, but their degradation process is generally slower than physicochemical methods. Several organisms, including plants, algae, fungi, bacteria, and other microbes, can break down the MP and remove it from the environment. While these groups of microorganisms are present, they also require other factors, including the type and number of pollutants, the pollution site, and other environmental factors such as temperature, pH, and the presence of electron acceptors and donors for the build-up of successful bioremediation treatment.^152,153^ Compared to other remediation techniques, bioremediation has several significant advantages for removing microplastics from the environment. It is a natural process and avoids producing toxic substances. *Ideonella sakaiensis* bacteria degrade the PET polymer but do not produce any type of toxic products. It is a natural process it takes a little time to remove the pollutants from the pollutant site.^154^ After decomposition, the byproducts produced, including cell biomass, water, and carbon dioxide, are commonly harmless.^155^ In comparison to other conventional technologies, it is an efficient process used for the cleanup of harmful toxic waste for the treatment of polluted areas. Through this remediation, toxic molecules are converted into less harmful products, and the toxic pollutants are completely reduced. Nutrients, particularly fertilizers, are supplied for supporting microbial growth to create fast and active microbial production. This process is easy, less expensive, and less labor-intensive. It is the current approach to cleaning the environment of major pollutants and providing sustainable, environmentally friendly opportunities.^156^ The efficiency of physical and chemical technologies for removing MPs from the environment has such challenges. Physical treatment processes such as membrane filtration, density separation, and adsorption have some drawbacks. Membrane-based filtration faces challenges because it cannot effectively capture particles that are less than 1 micron, this limits the overall efficiency of the method, especially for MPs.^157^ It is less sustainable for large-scale applications. The process of density separation for MPs has significant limitations. MPs have different densities that closely bind with organic materials, sediment, and other different inorganic particles, making it difficult to achieve effective separation. Fine MPs may not separate efficiently using a density-based method, and it is especially problematic for MPs that are difficult to separate from similar-density substances in the environment. It is less suitable for large scale field applications. Another treatment, adsorption process depends on the surface area of pollutants, pore size, and chemical composition of the adsorbent.^158^ When adsorbent materials are turned to the saturated phase, it can no longer remove the excess pollutant, restricting the period and extent of the removal process.^159^ So, this treatment requires excess adsorbents, which are more expensive. Different chemicals of the adsorbent may not always be sustainable or economically viable. Adsorption tends to be slower than other methods, particularly for fine microplastics and limiting its suitability for large-scale applications. Chemical treatments, such as coagulation, photocatalysis, and oxidation treatment processes, also present several significant challenges. Coagulation methods are highly dependent on the amount and types of coagulants, but they cannot efficiently remove all sizes and types of MPs, whereas photo catalysis and oxidation treatment process can break down MPs but require long-term time duration, after degradation toxic chemical substances are produced as a byproduct. It is a highly energy-consumption, concerns about the environmental impact, mainly large-scale application.

**Table 3:** Advantages and disadvantages of physical, chemical, and biological remediation of MPs

Name of the remediation method	Advantages	Disadvantages	Ref.
Membrane filtration	High removal efficiency	Less sustainable and more expensive	48
Density separation	Easy process, low cost	Less suitable, limited efficiency, time-consuming	9
Adsorption process	Eco-friendly, low-cost	More expensive, less sustainable	58
Coagulation and flocculation	Cost-effective	Produce the secondary pollutants	65
Photo catalysis	Environmental friendly under solar irradiation	Slow degradation, highly energy-consumption	82
Bioremediation	Sustainable and environmental friendly	Slow degradation	156

## 8. Alignments to SGD goals

Integrated approach of microplastic removal encompassing physical, chemical, and biological treatment techniques within a circular economy framework aligned with key Sustainable Development Goals (SDGs). Physical methods include membrane filtration, sand filtration, density separation, adsorption, each contributing uniquely to the capture or degradation of microplastics in various environmental media. Ultrafiltration membranes, such as polyether sulphone (PESP), have demonstrated removal efficiencies of 91–96% for nano-sized polyethylene (PE), polyvinyl chloride (PVC), and polyethersulfone (PES) particles.^42^ ZIF-8 aerogel composite membranes exhibit ∼91% removal for PVDF and polystyrene (PS).^44^ Using concentrated salt solutions such as NaI and NaH_2_PO_4_ achieves selective separation of microplastics with density-dependent efficiency but suffers reduced effectiveness for polymers like polyethylene terephthalate (PET).^53^ Moderate heat treatment can improve separation efficiency by increasing solution density.^53^ Chitin-graphene oxide sponges have shown excellent removal capacity for both microplastic particles and beads through a physical adsorption mechanism, while Zn–Al layered double hydroxide granules achieve up to 96% removal of nano-sized plastics.^60,61^ These processes primarily operate through size-based separation mechanisms and are capable of removing a suspended plastic particles from wastewater, drinking water. Consequently, physical remediation technologies contribute significantly to SDG 6 by improving water quality and minimizing the release of microplastics into fresh water and marine environments.

Chemical remediation techniques, such as coagulation–flocculation, photo-catalysis, and advanced oxidation processes (AOPs) are considered for degradation and removal of MPs. Coagulation with aluminium and iron chloride yields variable removal efficiencies, generally below 90%, limited by interactions with organic matter and pH conditions.^36^ Polymers like polyethylene (PE) removal improves dramatically with added AlCl_3_ coagulants.^160^ Advanced oxidation processes, employing TiO_2_- and ZnO-based photo catalysts, promote microplastic degradation and fragmentation, but quantitative efficiencies vary with polymer type and exposure, with partial mineralization producing CO_2_ and H_2_O after prolonged treatment.^72,76^ AOPs generate highly reactive radicals capable of oxidizing and fragmenting polymeric materials. These technologies support SDG9 (Industry, Innovation and Infrastructure) through the current wastewater treatment systems and sustainable industrial practices. These approaches, however, often face challenges such as efficiency limits for fine particles, energy consumption, and generation of secondary residues, which could reduce their environmental compatibility with SDG 13 objectives.^157^

Biological methods, including phytoremediation, bacterial, fungal, and microalgal degradation, leverage natural and engineered microbial and plant systems to break down microplastics into benign or value-added products like bioenergy, biomass, and reusable materials. This biological detoxification not only improves environmental health but also supports economic valorization of waste streams, thereby promoting sustainability.^161^ Several microorganisms and extracellular enzymes have shown the capacity to degrade polymers such as PE, PP, PS under controlled conditions. These biological approaches are strongly associated with SDG 14 and SDG 15 because they reduce ecological toxicity and contribute to the preservation of aquatic and terrestrial biodiversity. Bio-based adsorption, like biochar, activated carbon, and other natural adsorbents has low cost, eco-friendly, and resource-efficient characteristics.^62^ Such strategies further support SDG 12 by promoting sustainable resources utilization and circular economy principles. The circular economy outputs indicated, the emphasize transforming microplastic pollution into secondary resources, such as biofuels and biomaterials, closing the loop on waste management. Microbial communities facilitate the capture, adsorption and remediation of smaller plastic before their discharge into natural ecosystems. These systems provide environmentally sustainable and economically possible treatment possibilities while simultaneously supporting ecosystem restoration and urban sustainability. Future research should emphasize the development of energy-efficient, low-cost, and environmentally sustainable remediation systems with reduced secondary pollution to support SDG 6 (Clean Water and Sanitation), affordable energy (SDG 7), climate action (SDG 13). Furthermore, the integration of renewable energy sources, advanced biodegradable materials, synthetic enzymes, microbial consortia, and circular economy frameworks may significantly enhance the contribution of microplastic remediation technologies toward achieving global sustainability goals and long-term environmental protection under SDG 12 (Responsible Consumption and Production) while reducing ecological risks to aquatic and terrestrial ecosystems in alignment with SDG 14 (Life Below Water) and SDG 15 (Life on Land).^162^ The closed-loop design visually reinforces the continuous cycle of pollutant removal, waste valorization, and environmental restoration possible through integrating these diverse technologies.

## 9. Efficiency of biological treatment methods

While less quantified, phytoremediation has demonstrated considerable capacity to immobilize and degrade microplastics in wetland environments, aided by synergistic rhizospheric microbial communities.^161^ Specialized bacteria like *Ideonella sakaiensis* enzymatically degrade PET polymers into non-toxic products with efficiency reported between 60–80% under optimized laboratory conditions.^97^ Fungal enzymatic systems achieve significant depolymerization of various plastic types, although specific removal percentages remain under active investigation, with estimated degradation rates exceeding 50% over extended periods.^4,9^ Microalgae facilitate rapid removal of microplastics alongside water quality improvement, with removal efficiencies up to 95% demonstrated in controlled bioreactor studies.^110^ Taking advantage of these diverse efficiencies, engineered combinations of biological approaches can achieve broader, more consistent microplastic removal outcomes while optimizing resource recovery for circular economy models.^163^ Valorized biomass and purified water align with SDGs, enabling sustainable environmental management. Phytoremediation using microalgae and cyanobacteria has recently expanded as a sustainable approach for MP removal and degradation.^164^ Algal biomasses can adsorb, and aggregate MP particles through extracellular polymeric substances (EPS),^164^ facilitating their removal from aquatic environments. Some algal species produce oxidative enzymes and reactive oxygen species that contribute to partial polymer degradation.^165^ Coupling algal systems with wastewater treatment processes is considered a low-cost, carbon–neutral, and environmentally friendly strategy for large-scale MP remediation. Engineered biofilm reactors are currently being explored as sustainable large-scale technologies for improving microplastic remediation efficiency.^166^ Furthermore, advances in microbial genomics have proven that microbial communities can progressively modify their metabolic and enzymatic systems to exploit synthetic polymers as alternative carbon and energy sources in polluted ecosystem.^167^ Now adaptive microbial responses are now regarded as promising pathway toward improving the efficiency, sustainability, and large-scale applicability of biological MP remediation strategies.

## 10. Conclusion

This study summarizes several physico-chemical, biological methods for the removal of MPs along with thorough evaluation of advantages and disadvantages of each remediation technique. Although physio-chemical treatment is being used in very effective way to remove MPs, but these processes still produce harmful byproducts that are CO_2_, and H_2_O. The physical and chemical remediation methods effectively help for the removal of microplastics, but still have some challenges. Furthermore, a brief discussion of the mechanisms under the microbial-mediated breakdown of MPs, numerous MP-degrading useful microbes have been identified and isolated which can reduce the MP abundance in aquatic and terrestrial environments through the sustainable bioremediation process. So our study mainly focuses on the bioremediation process for removal of MPs sustainably, as this process is eco-friendly and pollution-free compared to other treatments which also support environmental sustainability and durability against plastic pollution. Future research should develop sustainable, eco-friendly, and cost-effective bioremediation technologies with higher degradation efficiency and lower environmental impacts. Researchers should also identify efficient microbial strains, produce synthetic enzymes, and design biotechnology systems for large-scale degradation of persistent microplastics. In addition, improving detection and monitoring techniques will help researchers better understand degradation mechanisms and enhance remediation efficiency. So, no single technology can completely remove microplastic pollution from ecosystem. Therefore, integrated treatment approaches combining physical, chemical, and biological processes are increasingly considered the most effective long-term strategy due to their enhanced removal efficiency and broader treatment capabilities.

## Ethics approval and consent to participate

No ethical approval has been required for the present study as of no such human, animal or biological samples were used directly.

## Consent for publication

The results/data/Figures in this manuscript have not been published elsewhere, nor are they under consideration by another publisher.

## Author contributions

S. C. U. D. and K. D.; conceptualization, methodology, data analysis, data curation, writing – original draft, writing – review & editing, visualization. S. C.; formal analysis, and data collection, U. D. and K. D.; supervision, P. K.; writing – review & editing, visualization.

## Conflicts of interest

The authors declare that they have no known competing financial and non-financial interests or personal relationships that could have appeared to influence the work reported in this paper.

## Data Availability

No primary research results, software or code have been included and no new data were generated or analysed as part of this review.
